# RNA Interference in Mammalia Cells by RNA-3’-PNA Chimeras

**DOI:** 10.3390/ijms9030299

**Published:** 2008-03-12

**Authors:** Nicoletta Potenza, Loredana Moggio, Giovanna Milano, Vincenzo Salvatore, Benedetto Di Blasio, Aniello Russo, Anna Messere

**Affiliations:** 1 Department of Life Sciences, Second University of Naples, Via Vivaldi 43, I-81100 Caserta, Italy; E-mail: nicoletta.potenza@unina2.it (Nicoletta Potenza); vincenzo.salvatore@unina2.it (Vincenzo Salvatore); aniello.russo@unina2.it (Aniello Russo); 2 Department of Environmental Sciences, Second University of Naples, Via Vivaldi 43, I-81100 Caserta, Italy; E-mail: loredana.moggio@unina2.it (Loredana Moggio); giovanna.milano@unina2.it (Giovanna Milano); anna.messere@unina2.it (Anna Messere))

**Keywords:** modified siRNA, RNA interference, RNA-PNA chimeras, Peptide Nucleic Acids, RNA

## Abstract

The discovery of siRNAs as the mediators of RNA interference has led to an increasing interest in their therapeutic applications. Chemical modifications are introduced into siRNAs to optimize the potency, the stability and the pharmacokinetic properties *in vivo*. Here, we synthesize and test the effects of RNA-3’-PNA chimeras on siRNA functioning and stability. We demonstrate that the chemical modifications are compatible with the siRNA machinery, because all the PNA-modified siRNAs can efficiently mediate specific gene silencing in mammalian cells. Furthermore, we find that the modification on the sense strand of siRNA results in an increased persistence of the activity, whereas modification on both strands results in enhanced nuclease resistance in serum.

## 1. Introduction

RNA interference (RNAi) is an endogenous gene-silencing mechanism triggered by double-stranded RNAs that mediates sequence-specific degradation or translational block of the target mRNA [1]. Small double-stranded RNAs (siRNAs) found in the cell are derived from the cytoplasmic processing of long dsRNA by the RNase-III type enzyme termed Dicer [2–3]. Dicer cleaves long dsRNA into approximately 21 nucleotide siRNA duplexes that contain 2-nucleotide 3′-overhangs with 5’-phosphate and 3′-hydroxyl termini. In a subsequent step, one strand of the siRNA is incorporated into the RNA-induced silencing complex (RISC) [4], which is then guided to cleave the target mRNA containing a perfectly complementary sequence through its Argonaute 2 protein component [5–6].

RNAi can be induced in mammalian cells by transfecting short (<30 nt) synthetic siRNAs which allow for sequence-specific gene silencing yet avoiding the non-selective toxic effects of long dsRNA [7]. Small interfering RNAs can also be expressed from vectors transcribing double-stranded hairpin-like RNAs (short hairpin RNA, shRNA) that are then processed into siRNAs inside the cell [8].

RNAi is proving to be a robust and versatile technique for down-regulating gene expression. In recent years, siRNA technology has found widespread use as a tool in functional genomics studies in cultured mammalian cells *in vitro*[9]. Since the demonstration of the efficacy of siRNAs in mammalian cells, these molecules have also been used to successfully target various infectious agents like human immunodeficiency virus [10], poliovirus [11], parainfluenza virus [12], respiratory syncytial virus [12], hepatitis B virus [13]. The majority of these studies suggests that, if properly designed, siRNAs might offer a fast, potent and easy option for therapy of many human diseases.

However, application of siRNAs *in vivo* and their possible use as therapeutics still face several critical hurdles, including siRNA delivery, bio-stability and pharmacokinetics. Many of these issues are not new to oligonucleotide-based therapeutic approaches, such as those based on antisense oligonucleotides, aptamers and ribozymes. Critical advances in this field have come from the development of nucleotide analogues with improved properties over natural nucleotides and recently several of these have been examined as a means to improve the prospect for siRNA therapy. One can envision improving the potency of siRNA with appropriate chemical modifications in terms of stability, target binding affinity, favoured conformational features of the modification such as RNA-like sugar pucker of the nucleoside (C3-endo) and A-helix geometry, altering on-rates and off-rates of hybridization and enhancing the product release. Several research groups have attempted to identify chemical modifications that increase the intracellular and extracellular stability of siRNAs by decreasing their susceptibility to nuclease attack, while allowing them to maintain sufficient gene-silencing activity for therapeutic use [14]. These studies have demonstrated that inhibition of gene expression by RNAi is compatible with a broad spectrum of chemical modifications, including terminal and internal modification [15], affording a wide range of useful options for probing the mechanism of RNAi and for improving RNA interference *in vivo*. The effects of chemical modifications are being evaluated and some rules are emerging, although a number of contrasting results have been published. Generally, modifications on 5’ half of the antisense strand are less tolerated than modifications on the 3’ half and the sense strand is more tolerating than the antisense strand [16–19]. Chemical modifications within the backbone of the oligonucleotide (phosphorothioate linkages P=S, boranophosphonate P=B) cause a small loss in binding affinity, but offer increased nuclease resistance, although some cytotoxic effects can be observed [17, 19–20]. Sugar modifications, such as 2’-H, 2’-F [18, 20], 2’-OMe [16], 2’-amine [21], 2’OMOE [22], 2’-OAl [17], LNA [22–23], ENA [24], FANA [25] and 4’-thio [26] are compatible with the RNAi machinery and in some cases they provide an enhanced nuclease resistance and improved activity. In contrast, all nucleobase modifications produced deleterious effects compared with the wild-type siRNA. Evaluation of chemical modification of the termini showed that only the modifications that did not block the 5’ end or retain the 5’-phosphodiester linkage of the antisense strand were tolerated [4, 27] whereas modification of the sense strand had no effect on silencing activity [16, 18]. In contrast, the available data regarding modification of the 3’ terminus of the siRNA are not conclusive, because some modifications are tolerated (inverted deoxy abasic residue or an amino group at the 3’ end of both strands; puromycin, biotin, ddC or aminopropyl group at the 3’ end of the antisense strand), but other modifications (e.g., 2-hydroxyethylphosphate, 2’-O,4’-C-ethylene thymidine at the 3’ end of the antisense strand) result in a loss of activity [19]. Certain terminal conjugates (cholesterol, lithocholic acid, lauric acid or long alkyl branched chains, TAT peptide)[28–29] have been reported to improve cellular uptake without perturbing gene silencing activity. With a few exceptions, increasing degree of modification along siRNAs impairs their activity. While this may be compensated by the nuclease stability and/or specifity imparted by some oligonucleotide chemistries, the prediction of effective siRNA chemistries remains an active focus of continued study. An improvement in the intracellular and extracellular stability of the siRNA is still one crucial problem for the successful *in vivo* application of the synthetic siRNA.

Peptide Nucleic Acids (PNAs) are oligonucleotide mimics in which the sugar-phosphate backbone has been replaced by a pseudo-peptide backbone [30]. When used in antisense constructs, PNA confers chemical and enzymatic stability and high affinity towards complementary DNA and RNA [31–32]. Nonetheless PNA have limited solubility and tendency to aggregate and are not easily internalized into cells, whereas DNA-PNA conjugates resulted in molecules with higher solubility and increased capacity to cross biological membranes as compared to canonical PNA. Chimeric molecules in which tracts of DNA are bound to N and/or C terminus of PNA have been widely reported [33–37]. Furthermore, some parameters, as for example PNA/DNA bases ratio, the sequence and the site of conjugation (whether it is 5’ or 3’ junction) can be varied to modulate thermal stability of the chimera/DNA(RNA) duplexes [33]. In contrast to DNA-PNA chimeras, only few studies have been conducted on chimeras constituted by RNA-PNA. One example is given by a (2’-O-methyl-RNA)-3’-PNA chimeras which resulted of interest as potential antisense agent [38]. So far, no results have been reported for the use of RNA-PNA chimeras in RNAi, even if the advantage of mixing peptide bonds and nucleic acids bond has been demonstrated [28, 39].

These studies prompted us to join the benefits of the PNA technology with the potency of the RNAi applications. In particular, we decided to investigate the effects on the RNAi activity of a thymine dimer composed of PNA monomers in the 3’-overhanging tract of RNA (RNA-PNA chimeras).

In the present work, we report the synthesis of siRNAs composed of RNA-PNA chimeras, their chemical-physical characterization and the evaluation of their gene silencing activity in cultured mammalian cells, based on their ability to target the firefly luciferase mRNA. UV melting profiles and CD spectra of chimeric siRNAs showed that the introduction of the PNA units on either or both strands had no effect on thermal stability and conformational features of the double-helical molecule. Next, we demonstrated that the chemical modifications were compatible with the siRNA machinery and showed that the modification on the sense strand produced an increased persistence of the silencing activity, whereas the modification on both strands yielded an enhanced serum stability. These results provide a basis for the development of further PNA-based siRNAs to probe the molecular mechanism of RNAi and to improve their chemical and functional properties for *in vivo* applications.

## 2. Results and Discussion

### 2.1 Synthetic strategy and characterization of native and chemically modified siRNAs

We decided to use for our studies an effective siRNA targeting firefly luciferase mRNA described elsewhere [7]. Automated luciferase assays allowed us to judge easily and accurately the gene silencing efficiency of chemically modified siRNAs in comparison to the native siRNA.

**Figure 1.**
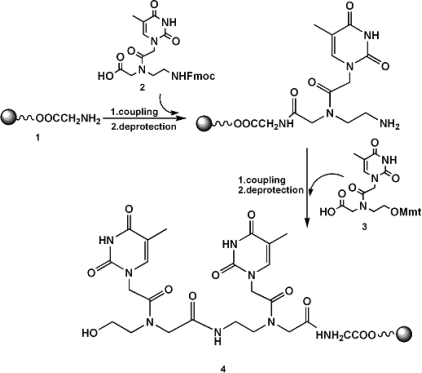
Coupling cycles for the assembling of PNA dimer in 3’-end of RNA oligomers.

We synthesized sense and antisense strands of wild-type siRNA ^5’^UCGAAGUAUUCCGCGUACGTT^3’^ and ^5’^CGUACGCGGAAUACUUCGATT^3’^ and of modified siRNA in which the 3’-TT ovherangs are substituted by an overhanging tract of PNA (RNA-3’-PNA chimeras). Wild-type siRNAs were synthesized following fully automated method for DNA/RNA synthesis. The fully automated synthesis of the chimeras ^5’^UCGAAGUAUUCCGCGUACG^3’^ttGly (sense) and ^5’^CGUACGCGGAAUACUUCGA^3’^ttGly (antisense) was carried out on a glycine functionalized CPG-OH support using different PNA monomers (Fmoc-amino and MMT-hydroxy-aminoethylglycine and commercially available phosphoramidite ribonucleotides) following standard PNA and RNA chemistry (Figure 1).

It has been previously described [38] that the binding affinity of a DNA-3’-PNA chimera to a complementary DNA sequence is higher when the 3’-part of the oligodeoxynucleotide is linked to a terminal hydroxy-aminoethyl-glycine unit of the PNA via a phosphodiester linkage. Since the type of junction in the DNA-PNA chimeras appeared to significantly influence binding affinity in the duplexes formation [40], we aimed at applying the same linking moiety at the junction of RNA and PNA by using the PNA-DNA linker molecule **3** (Figure 1). In order to incorporate the thymine PNA dimer at 3’ ends of RNAs, commercially available PNA monomer **2** and O-monomethoxytrityl (Mmt) derivative **3** (Figure 1), which was synthesized by the reported procedure [41], were assembled on a CPG support.

Therefore, the solid support CPG-OH resin was functionalized with a Fmoc-Gly-OH using PyBop/HOBT as activating agents. Chain elongation of PNA dimer proceeded on support **1** as showed in Figure 1 alternating Fmoc-amino and MMT-hydroxy-ethylglycine PNAs (**2** and **3** respectively in Figure 1). First, PNA monomer **2** was coupled using PNA standard procedure. After deblocking of amino function, Mmt-O-PNA monomer **3** was coupled. The coupling cycle for modified PNA monomer was carried out by a slightly modified protocol. After deprotection of OH function, the synthesis of RNA tract of chimera was performed on support **4** (Figure 1) using commercially available phosphoramidite ribonucleotides on a RNA synthesizer. All the synthesized oligomers (21-mers) were purified by HPLC and characterized by MALDI-TOF mass spectrometry. The MS-data confirmed the identities of the synthesized oligomers.

Then, the oligomers were combined to form siRNAs in the four possible combinations (Table 1): siRNA A, with unmodified sense and antisense strands; siRNA B, with modified sense strand and unmodified antisense strand; siRNA C, with unmodified sense strand and modified antisense strand; siRNA D, with modified sense and antisense strands.

Thermal stability of the siRNA A, B, C and D was studied by thermal denaturation UV experiments showing an identical thermodynamic stability of the duplexes. In fact, all the duplexes exhibited the same Tm (80°C) suggesting that the presence of PNA dimer linked through a natural phosphodiester junction in 3’ overhangs of siRNA caused no destabilization of the chimeric siRNA, as inferred from UV-melting profile.

**Figure 2.**
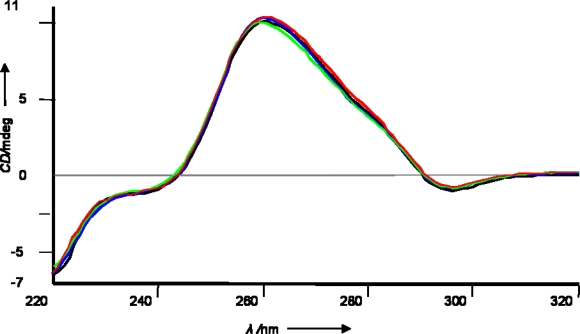
CD spectra of siRNAs [1.0 μM]. A (black), B (red), C (blue), and D (green). The spectra were recorded in RNAse-free buffer.

Furthermore, but not surprisingly, the modified duplex were found to retain conformationally RNA-like A-type helical characteristics. In fact, the CD spectra of the duplexes containing RNA-PNA chimeras (B, C, D), recorded in the same buffer used for UV melting experiments, were identical to the spectra of the canonical siRNA A, showing a typical positive shoulder at 280 nm and a large positive band at 260 nm (Figure 2). These data show that the 3’-end chemical modification does not interfere with the typical A conformation of a dsRNA. Even though, conformational features alone do not decide the interfering activity, a RNA-like A conformation is required for effective gene silencing [18].

### 2.2 Compatibility of RNA-3’-PNA chimeras with the siRNA machinery

**Figure 3.**
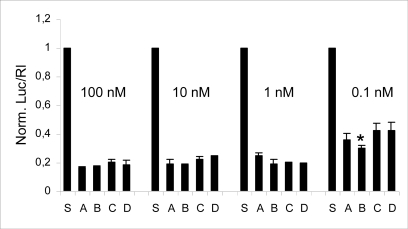
RNAi activity of native and modified siRNAs at inhibiting luciferase expression in HeLa cells. The firefly luciferase activity (luc) was normalized to the Renilla luciferase activity (Rl) and the uninhibited activity (plasmids encoding the luciferases co-transfected with scrambled siRNA, S) was set to 1. Data represent mean normalized luciferase activity from at least three experiments ± s.d. The transfected siRNA concentrations are indicated. * p <0,05, as detailed in the text.

To verify the compatibility of PNA units into siRNA with the RNAi machinery, we have evaluated the different siRNAs for their ability to specifically inhibit firefly luciferase in HeLa cells. 100 nM siRNA duplexes were co-transfected with the reporter plasmid combination pGL2/phRL-TK into HeLa cells using cationic liposomes. pGL2 encodes the target *Photinus pyralis* luciferase and phRL-TK encodes the *Renilla reniformis* luciferase used as control to normalize data with the efficiency of transfection. Luciferase activities were determined 48 h after transfection. As shown in Figure 3, the native siRNA (siRNA A) effectively and selectively reduced firefly luciferase activity by more than 80% as previously reported [7], whereas an unrelated siRNA (scrambled siRNA, S) was virtually ineffective. Introduction of PNA modifications in the 3’ overhang in either or both strands revealed no loss of inhibitory effect, because all the modified duplexes were as efficient as the native siRNA. These results indicate that the PNA units into 3’-dangling dinucleotides (dTdT) are well tolerated by RNAi machinery in the sense strand, in the antisense strand and in both strands of the siRNA. Dose response data indicated that decreasing the siRNA concentration from 100 to 1 nM did not reduce the specific silencing effect of the modified siRNA; it decreased to about 60% only if the siRNA concentration was dropped to 0.1 nM. At this concentration, the siRNA B appeared to be slightly but significantly, more effective than the other modified duplexes (t-test value of p < 0.05 for comparison with both siRNA C and D). This is most likely due to the better toleration of sense strand to chemical modification in comparison to the antisense strand that is retained by RISC and used as a guide for target mRNA recognition.

### 2.3 Persistence of modified siRNA-mediated silencing

**Figure 4.**
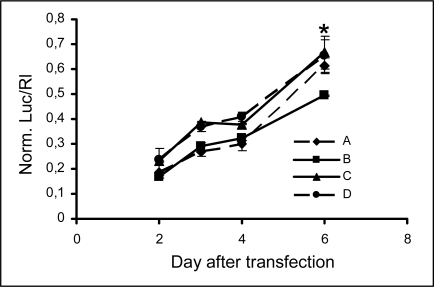
Persistence of luciferase silencing by native and modified siRNA. Stably expressing luciferase HeLa cells were transfected with 25 nM siRNA. Luciferase activity was measured 2, 3, 4 and 6 days post-transfection and reported as time-course curves for each duplex. Data represent mean normalized luciferase activity from at least three experiments ±s.d. compared with the control siRNA set at 1. * p<0,01, as detailed in the text

The effect of siRNA in cultured mammalian cells is transient and typically lasts few days. The ability to extend the period of effective silencing would be very important for a possible use of siRNA in therapy. Thus, to further characterize the inhibitory properties of the RNA-3’-PNA chimeras, we decided to examine the duration of their activity in comparison with the unmodified siRNA. To this purpose, we produced HeLa cells stably expressing the target reporter gene by co-transfecting pGl2 plasmid along with pcDNA 3.1 plasmid carrying the gene for the resistance to G418. After two weeks of selection, G418 resistant clones were obtained. The screening for the expression of luciferase gene yielded luciferase stable transfectants which were successively used for the time-course experiments. Cells were transfected with 25 nM siRNAs, and the luciferase expression was monitored after 2, 3, 4 and 6 days (Figure 4). Luciferase suppression lasted over the 4 day period with only a slight decreasing in the inhibitory effect. Six days after transfection the inhibitory effect decreased to about 40% and some differences among siRNA activities became evident. The siRNA B activity appears to be more persistent than that of the other duplexes and these differences are statistically significant. In fact, the t-test for comparison between siRNA B activity and siRNA A activity resulted in a p value < 0.0005 and the comparison between siRNA B activity and siRNA C or D activity resulted in a p value 0.0085 and 0.017, respectively. In other words the persistence of the silencing effect over a 4-day period is similar for duplexes C and D on one hand and A and B on the other, whereas, at 6 days from transfection, the siRNA B retains a potency that is higher than that of native siRNA. This effect may be due to a faster depletion of the intracellular siRNA pool of unmodified siRNA.

### 2.4 Serum stability of modified siRNA

**Figure 5.**
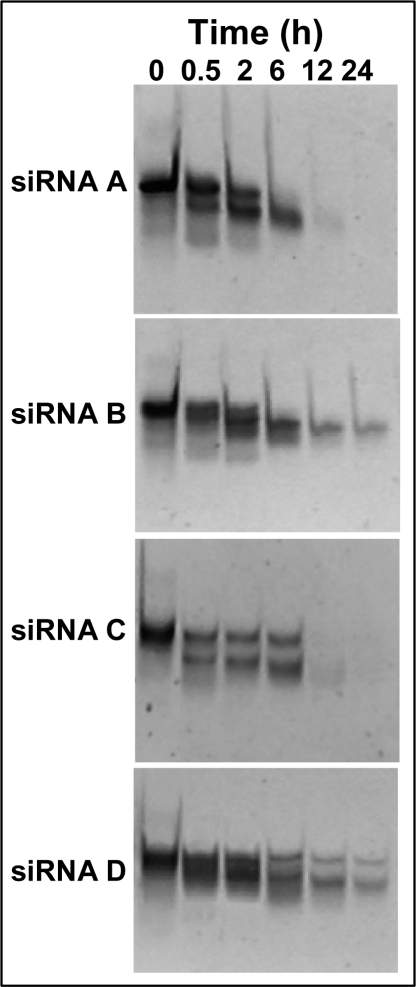
Serum stability of native and modified siRNAs. The various siRNAs were incubated in FBS at 37 °C for the indicated times and analyzed by PAGE ethidium bromide staining. The panels show the results of one representative experiment out of at least two independent ones.

Having inquired the intracellular stability of the modified siRNAs, experiments were planned to test whether the introduction of PNAs in 3’ end could lead to a siRNA that is more stable in the extracellular environment. In this regard, it has been previously shown that PNA substitutions enhance DNA resistance to serum-derived nucleases [34]. To investigate whether the introduction of the PNA units into siRNA would provide similar enhancement of nuclease resistance, siRNAs were incubated in fetal bovine serum at 37 °C. After various times, aliquots of each siRNA were analyzed by electrophoresis on 20% polyacrylamide gels to detect any degradation products (Figure 5). As expected, the unmodified siRNA was rapidly degraded. A similar degradation profile was observed for the siRNA C; however, this duplex was more stable than siRNA A, because after 6 h of incubation an electrophoretic band corresponding to the intact duplex was still evident. The siRNA B showed an intermediate degradation rate. The introduction of PNA dimer at the 3’ end of both strands increased markedly the resistance to serum-derived nucleases; in fact the siRNA D showed a significant improvement in stability, because a band corresponding to the intact duplex was still evident even after 24 h of incubation.

### 2.5 Discussion

SiRNAs represent a new tool for functional knock-down studies in mammalian cells. In order to facilitate their use as potential therapeutic nucleic acids, much effort has been put into identifying chemical modifications that could improve their potency, stability and pharmacokinetic properties *in vivo* [14].

In this study, we modified the backbone structure of a siRNA targeting the mRNA of firefly luciferase, a reporter enzyme whose expression in mammalian cells was used to assess the efficacy of the RNAi. Generally, each strand of the siRNA duplex is 21 nucleotides long, and designed with two nucleotides 3’-overhangs. This structure is the natural one deriving from ATP-dependent processive cleavage of long dsRNAs by Dicer [2–3]. Synthetic siRNAs usually have two deoxy-thymidines at the 3’ termini of both strands (the ‘Tuschl Design’). We decided to include modifications only in the non-basepairing 3’-overhangs, which were known to better tolerate various types of modifications. On the other hand, it has been reported that the nature and base stacking of a 3’-double-nucleotide overhangs can make a significant contribution to the thermodynamic stability of RNA duplexes and could have a role to enhance the distinction between the two ends of the siRNA duplex for the strand selection and loading into the RNAi effector complex [42–43].

We substituted the two thymidines at the 3’-termini with a PNA thymine dimer at either or both strands of the siRNA. PNAs are widely used as antisense, antigene molecules and as biotechnological tools due to their chemical and enzymatic stability and to their high affinity towards complementary DNA and RNA [31–32]. Conjugation of DNA to PNA have been widely described. The biological activity of these chimeric molecules revealed very appealing; in fact, in contrast to PNAs, they are soluble in aqueous media, are able to recognize exclusively in antiparallel fashion single strands of DNA and RNA; when used in antisense, PNA-DNA chimeras can trigger Rnase H-mediated mRNA degradation more efficiently compared with the antisense DNA in DNA/RNA substrates [33]. Furthermore, they are more stable than DNA to nucleases degradation. In contrast to DNA-PNA chimeras, very little investigation has been reported on chimeras constituted by RNA-PNA and no results have been still reported for their RNAi application.

In this work, we synthesized the modified siRNAs and assayed them to establish their RNAi activity in mammalian cells and their intracellular and extracellular stability. We showed that the introduction of PNA in the 3’-overhangs in either or both strands of siRNA are well tolerated by the RNAi machinery, because all the modified siRNAs still mediated efficient gene silencing. PNA is a dramatic departure from RNA because of its modified amide backbone. However, our results show that the introduction of PNA units into siRNAs did not interfere with the unwinding of the siRNA duplex, incorporation of the siRNA into RISC and/or the rate of target cleavage and product release. When the concentration of transfected siRNAs was dropped to 0.1 nM, siRNA B exhibited an inhibition activity higher than that of the other duplexes. This is probably due to the better toleration of sense strand (passenger strand) to chemical modification in comparison to the antisense strand (guide strand) that is incorporated into RISC and mediate the binding with target mRNA and mRNA cleavage. However, the siRNA C in which antisense strand was modified, and siRNA D, in which sense and antisense strands were modified, still showed a inhibition activity, slightly lower than the native siRNA A.

Thus, the duplex B is the best, perhaps because, for this duplex, RISC is preferentially loading the antisense strand that results the only strand with a normal overhang.

Overall, these results suggest that RISC can recognize the termini of the modified oligomers C and D, even if the modification regards the sugar-phosphate RNA backbone.

Thus the substitution of the phosphodiester linkage in the dinucleotide (T-T) with the amide, bond of the PNA dimer (t-t) in 3’-overhangs of antisense strands is compatible with the binding and the distortion into the conserved hydrophobic cleft between the β barrel and the PAZ –specific ββαmodule of Ago2 PAZ domain. These results are significant as a source of insights into the details of RNA recognition by siRNA machinery.

The structural basis for 3’ overhangs recognition is provided by the structures of PAZ in complex with single-stranded RNA (dAgo2 PAZ) [44] and for putative guide strand of duplex siRNA (hAgo1PAZ) [45]. The two nucleotides at the 3’-end retain a stacked A-form conformation, with the 3’-terminal residue buried deep within the pocket and targeted by numerous protein interactions. The non-bridging oxygens of the phosphodiester backbone linking the last two residues make four hydrogen bonds with Y309, Y314 and H269 and a water molecule stabilized by Y277. The sugar ring of the terminal residue is further anchored in place by van der Waals packing against L337 and T335, whereas its 2‘- and 3’-hydroxyl groups form hydrogen bonds to the backbone amide and carbonyl groups of Y336, respectively. In addition, portions of the sugar ring and base of the terminal nucleotide are stacked over aromatic residue F292. In our case, these same hydrogen bonds cannot occur because the sugar-phosphate backbone has been completely replaced by a pseudo-peptide backbone of the PNA dimer but it is likely that the oxygens or the nitrogens of the amide backbone of the PNA units could play as the donor or the acceptor of hydrogen bonds with residues of the 3’-end pocket.

One major drawback of the usage of siRNAs is its transient nature. Using conventional transient transfection protocols gene expression is inhibited for a maximum period of 2–4 days. Therefore, it will be beneficial to develop more stable siRNA molecules, especially for potential *in vivo* application, where sustained delivery is one major obstacle. An improvement in the intracellular and extracellular stability of the siRNA is still one crucial problem for their successful *in vivo* application.

In order to assess the duration of activity of modified siRNAs vs unmodified siRNA, we established a HeLa cell line stably expressing the target reporter gene. Monitoring the luciferase expression after a single transfection with the different siRNAs showed that for a longer period of time the siRNA B exhibited an higher potency over the other duplexes, following the same trend obtained in the dose-effect experiments (Figure 3). At this point we do not know whether this increase in stability will also improve the pharmacodynamic properties of these molecules, but these data suggest that an increased intracellular stability of RNA-3’-PNA modified sense strand siRNA may be responsible, at least in part, for the persistent activity of siRNA B.

However, when we examined the extracellular stability, the siRNA D showed the highest resistance against serum-derived nucleases, because 24 h after incubation an electrophoretic band corresponding to an intact duplex was still evident. Since siRNA must remain intact to interact with the RISC complex, it is likely that the extreme stability of this duplex together with a RNAi activity comparable to unmodified siRNA makes an important contribution to the application of RNAi for therapeutic use. In fact, it is important to be aware of various design principles, balancing the effects of the modifications on stability against the negative effects on activity.

In summary, we synthesized RNA-PNA conjugated molecules that retain RNA-like character but with new and favorable properties over unmodified siRNA. In fact, we can conclude that the RNA-3’-PNA chimeras are compatible with the siRNA intracellular machinery achieving an inhibition at concentrations up to 0.1 nM for sense strand modified siRNA. Moreover, we proved both conserved initial activity and, for siRNA with modified sense strand, an increased duration in time-course experiments. Notably, the introduction of a modest modification such as the substitution of only the 3’-overhanging dinucleotide with the PNA dimer on both strands of siRNA leads to significant resistance against serum-derived nucleases without loss of RNAi activity.

Our results provide a basis for the further development of synthetic PNA-RNA siRNA molecules with improved properties, including higher resistance to cellular and extracellular medium, and thus may impact positively on the use of chemically modified siRNAs in RNAi technology and broaden the perspective of translating the technology into a drug paltform. Moreover, these molecules represent a further tool for insights into the studies aimed to know new details on RNA recognition by siRNA machinery.

## 3. Experimental Section

### 3.1 Synthesis of RNA-3’-PNA chimeras

Controlled Pore Glass (CPG)-OH resin (LinK Technology) (loading 0.11 meq g^−1^) was used to performe the PNA dimer synthesis. The support was previously functionalized with a Fmoc-Gly-OH (Novabiochem) using PyBop/HOBT as activating agents (Novabiochem) to a final loading of 0.08 meq g^−1^. PNA dimer elongation proceeded on support **1** as showed in Figure 1 alternating Fmoc-amino (ASM Research chemicals) and MMT-hydroxy-aminoethylglycine PNA (**2** and **3** respectively in Figure 1).

Commercially available (2-aminoethyl)glycine-based PNA monomer **2** was used without further purification. N-[2-[(4-methoxyphenyl)-diphenylmethoxy]ethyl]-N-[(thymin-1-yl)acetyl]glicine PNA monomer **3** was synthesized as previously described [41].

Briefly, PNA monomer **2** (10 eq) was coupled on support **1** using PNA synthesizer (Expedite^™^ 8909 Nucleic Acid Synthesis System ABI) and standard solutions and protocols for automated synthesis of PNA. After deblocking of amino function by a solution of piperidine in DMF (20%), Mmt-O-PNA monomer was coupled by a solution 0.2 M PNA monomer **3** in ACN/DMF (1:1, v/v), 0.2 M DIPEA and 0.3 M lutidine in ACN/DMF (1:1, v/v). The coupling cycle for modified PNA monomer was carried out by a slightly modified procedure consisting in the following steps: 1) washing with 2.5 mL of (ACN/DMF) (1:1, v/v); 2) coupling 20 minutes; 3) washing with 2.5 mL of ACN/DMF (1:1, v/v); 4) capping with 5% of acetic anhydride and 6% of 2,6-lutidine in DMF, 2.0 mL; 5) washing with 2.5 mL of ACN/DMF (1:1, v/v) and 2.5mL of DMF; 6) deblocking with a solution of dichloroacetic acid (2%) in ACN; 7) washing with 2.5mL of DMF and 5mL of ACN. All reagents were purchased at Sigma-Aldrich.

At the end of the synthesis of PNA dimer, the resulting support **4** (0.035 meq g^−1^) was washed with ACN and used for the synthesis of RNA tract.

Ribonucleotide tracts were synthesized using 2′-OTBS-RNA phosphoramidite units (TBS = *t*-butyldimethylsilyl) commercially available (Link Technologies). The synthesis was carried out with an Applied Biosystems DNA/RNA synthesizer (Model Expedite) following standard procedures for RNA oligomers synthesis. The synthesis of unmodified RNA oligomers, deprotection, cleavage from support of all oligomers were performed following reported procedures [35, 46].

### 3.2 Purification and analysis of RNA-3’-PNA chimeras

After the cleavage from the support, the crude solution was filtered, concentrated and the chimeras were purified by anion-exchange HPLC (Nucleogel SAX column, Macherey-Nagel, 1000-8/46, Waters 600 Controller instrument, equipped with a Waters 996 photodiode array detector and Millennium software). The oligomers were eluted with a gradient from 0 to 100% B in A in 30 min, flow rate 0.8 mL/min, λ=260 nm; A: 20 mM KH_2_PO_4_, pH 7.0, 20% (v/v) ACN; B: 1M KCl, 20 mM KH_2_PO_4_, pH 7.0, 20% (v/v) ACN. The desalting was performed on a Sephadex-G25 column (NAP columns, Amersham Biosciences Inc.) eluted with water.

The identities and purities of purified chimeras were assessed by RP-HPLC (Waters, C-18, 3.9 × 300 mm, solvent A = 100mM trietylammonium acetate, pH 7, solvent B = ACN, 5–60 % B in 40 min, flow rate 1.5 ml/min, λ = 260 nm) and by mass spectrometry using a MALDI-TOF micro LR instrument Waters Micromass Co, with sinapic acid as matrix. The yields are indicated in parentheses as OD units at 260 nm starting from 0.2 μmol scale.

Modified sense RNA strand: t_R_=26 min; (OD=7.2). MS(MALDI-TOF) positive mode: m/z 6705 calcd for C_240_ H_255_ N_78_ O_145_ P_19_; 6783 found [M+2K]^+^.

Modified antisense RNA strand: t_R_=27 min; (OD=4.8). MS(MALDI-TOF) positive mode: m/z 6728 calcd for C_205_ H_256_ N_81_ O_143_ P_19_; 6806 found [M+2K] ^+^.

### 3.3 siRNA preparation, Tm measurements and CD spectra

Oligomers were resuspended in RNase-free annealing buffer (100 mM potassium acetate, 30 mM Hepes-KOH pH=7.4, 2 mM magnesium acetate) and equimolar ratios of the sense and antisense strands were annealed to form the duplexes to a final concentration of 25 μM by incubation at 90°C for 1 min and gradually cooling to room temperature.

The concentrations were estimated spectrophotometrically at 90° C using the following additive molar extinction coefficient ε260(L cm^−1^mol^−1^) T=8800, A=15400, C=7200, G=11500 and U=9900 for the natural nucleobases and t=8600 for the PNA monomers.

Melting curves of the siRNAs (1.0 μM) were acquired on a Jasco V-550 spectrophotometer equipped with a Jasco ETC-505T Peltier temperature programmer using a 1cm path-length quartz cell. Melting curves were recorded at 260 nm using a heating rate of 0.5°C/min, a slit of 2 nm and a response of 0.2 s. Tm values were obtained from the maxima of the first derivatives of the melting curves.

Circular Dichroism spectra of the siRNA samples (1.0 μM) were recorded at 15 °C using a 1 cm quartz cell in the Jasco J-815 spectropolarimeter equipped with a PFD-425S thermal controller unit.

### 3.4 Cells cultures and transfections

HeLa cells were grown at 37°C, 5% CO_2_ in Dulbecco’s modified Eagle’s medium supplemented with 10% Fetal Bovine Serum (FBS) (EuroClone), 100 units ml^−1^ penicillin and 100 mg ml^−1^ streptomycin (EuroClone). Cells were regularly passaged to maintain exponential growth. The day before transfection, cells were trypsinized, diluted in the appropriate amount of growth medium without antibiotics and transferred to 12-well plates (1 ml per well) such that they were at 80–90% confluent at the time of transfection. Co-transfections of reporter plasmids and siRNAs were carried out with Lipofectamine 2000 (Invitrogen) as described by the manufacturer. Per well 1 μg pGL2-Control (encoding *Photinus pyralis* luciferase), 0.05 μg phRL-TK (encoding *Renilla reniformis* luciferase) (Promega) and different concentrations of siRNA, formulated into liposomes, were applied. The cells were incubated with the transfection mix for 6 h and the medium was then replaced with new fully supplemented culturing medium. Luciferase activities were monitored 2 days after transfection.

Generation of HeLa cells stably expressing the firefly luciferase encoded by pGL2-Control was obtained as follows. HeLa cells were maintained in 60 mm dishes and transfected with 3.1 μg pGL2-Control and 0.4 μg pcDNA3.1 (Invitrogen) (encoding neomycin resistance gene) using Lipofectamine 2000. 24 hours after transfection, cells were diluted at a 1:10 into fresh growth medium and transferred to 10 cm dishes. The following day, G418 (Euroclone) was added to have a final concentration of 1 mg/ml to select for stable transfectants. Selective medium was replaced every 3–4 days. Resistant clones were harvested as a pool after 2 weeks, expanded and luciferase expression was monitored.

HeLa cells stably expressing luciferase gene were transfected with 25nM siRNA and 0.05μg phRL-TK using Lipofectamine 2000 for time-course experiments. Medium was replaced every second day after transfection.

### 3.5 Luciferase activity assay

Luciferase activity assays were performed using Dual-Luciferase Reporter Assay System (Promega) according to the manufacturer’s protocol. Briefly, cells were washed with phosphate buffered saline (PBS) and lysed. Cell lysates were centrifuged to remove cellular debris; 20 μl aliquots of each sample were placed into luminometer tube, followed by sequential auto-injection of 100 μl Luciferase Assay Reagent II (substrate for firefly luciferase) and 100 μl Stop and Glow Reagent (stop solution for firefly luciferase and substrate for Renilla luciferase). The mean of the luciferase activities measured for 10 s each were used to calculate ratios between firefly and Renilla luciferase.

### 3.6 Serum stability

7.5 μl of unmodified and modified siRNAs (25μM) were incubated in 100 μl of FBS at 37°C. Aliquots of 21.5 μl were withdrawn at different time points and immediately frozen. The solutions were then extracted with phenol and siRNAs were precipitated with ethanol. Samples were subjected to electrophoresis in 20% polyacrilamide-Tris-Borate-EDTA (TBE) under non-denaturing conditions and visualized by ethidium bromide staining. Equal amounts of siRNAs before serum incubation were extracted with phenol in parallel and loaded as an input control.

## Figures and Tables

**Table 1.** The sense and antisense strands of siRNA targeting firefly luciferase mRNA.

**A**	^5’^UCGAAGUAUUCCGCGUACGTT(sense)
	^3’^TT AGCUUCAUAAGGCGCAUGC(antisense)
**B**	^5’^ CGAAGUAUUCCGCGUACGttGly (sense)
	^3’^TT AGCUUCAUAAGGCGCAUGC(antisense)
**C**	^5’^ UCGAAGUAUUCCGCGUACGTT(sense)
	^3’^Glytt AGCUUCAUAAGGCGCAUGC(antisense)
**D**	^5’^ UCGAAGUAUUCCGCGUACGttGly(sense)
	^3’^Glytt AGCUUCAUAAGGCGCAUGC(antisense)
